# The miR393-Target Module Regulates Plant Development and Responses to Biotic and Abiotic Stresses

**DOI:** 10.3390/ijms23169477

**Published:** 2022-08-22

**Authors:** Jinjin Jiang, Haotian Zhu, Na Li, Jacqueline Batley, Youping Wang

**Affiliations:** 1 Jiangsu Provincial Key Laboratory of Crop Genetics and Physiology, Yangzhou University, Yangzhou 225009, China; 2 School of Biological Sciences, University of Western Australia, Perth, WA 6009, Australia; 3 Joint International Research Laboratory of Agriculture and Agri-Product Safety, The Ministry of Education of China, Yangzhou University, Yangzhou 225009, China

**Keywords:** miR393, target gene, plant development, stress response, auxin

## Abstract

MicroRNAs (miRNAs), a class of endogenous small RNAs, are broadly involved in plant development, morphogenesis and responses to various environmental stresses, through manipulating the cleavage, translational expression, or DNA methylation of target mRNAs. miR393 is a conserved miRNA family present in many plants, which mainly targets genes encoding the transport inhibitor response1 (TIR1)/auxin signaling F-box (AFB) auxin receptors, and thus greatly affects the auxin signal perception, Aux/IAA degradation, and related gene expression. This review introduces the advances made on the miR393/target module regulating plant development and the plant’s responses to biotic and abiotic stresses. This module is valuable for genetic manipulation of optimized conditions for crop growth and development and would also be helpful in improving crop yield through molecular breeding.

## 1. Introduction

Small RNAs (sRNAs) are 20–30 nucleotide (nt) noncoding RNAs abundant in plants and animals. They were first reported in *Caenorhabditis elegans* [1], and classified into microRNAs (miRNAs), small interfering RNAs (siRNAs), and piwi-interacting RNAs (piRNAs) [2]. The 20–24 nt miRNAs influence plant growth and development, secondary metabolism and response to biotic and abiotic stresses through transcriptional and translational repression of specific target genes with complementary sites [3,4]. miRNAs can also silence target genes through DNA methylation and histone modification [5]. miR393 is a conserved miRNA family identified in many plant species which targets the genes encoding auxin receptors, Transport Inhibitor Response1 (TIR1) and Auxin Signaling F-box (AFB), as well as basic helix-loop-helix (bHLH), thus affecting the homeostasis of auxin signaling and regulating plant development and different stress responses [6,7,8,9]. Here, we review the molecular roles of miR393 and the *TIR1/AFB Auxin Receptor* (*TAAR*) gene family, as well as other putative target genes in plant growth, development, metabolism, and stress responses.

## 2. miR393 and the Target Genes Are Strongly Conserved in Plants

**Figure 1:**
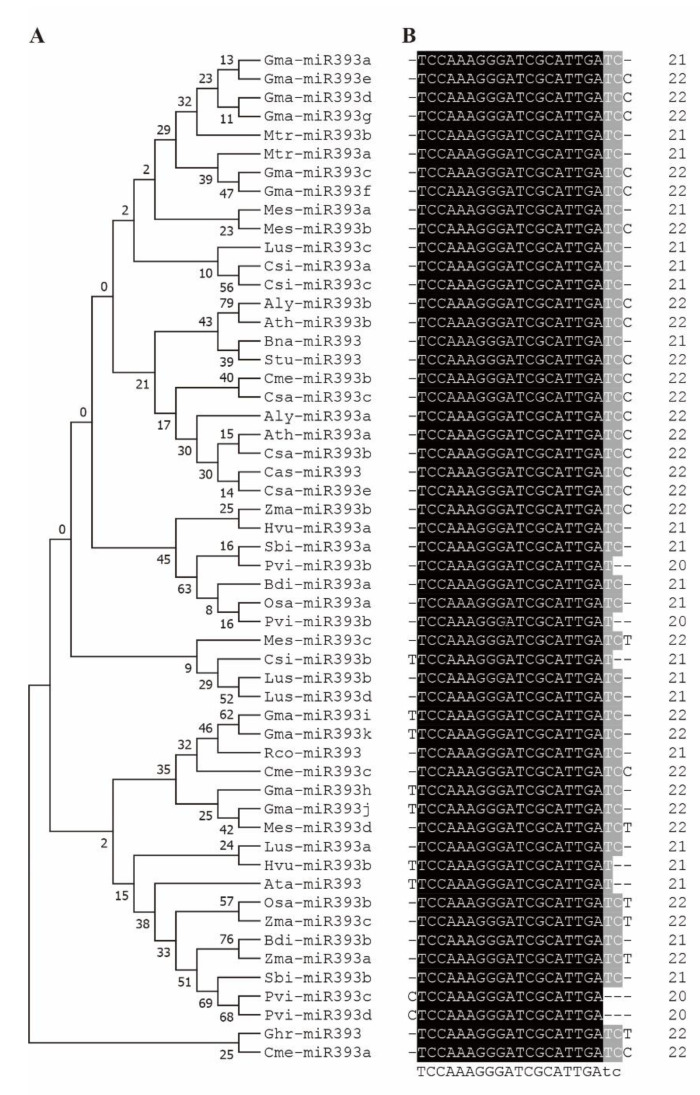
The conserved sequence similarity of miR393 members in different plants. (**A**) Phylogenetic relationships of pre-miR393s; (**B**) sequence similarity of mature miR393s. Aly, *Arabidopsis lyrata*; Ata, *Aegilops tauschii*; Ath, *Arabidopsis thaliana*; Bdi, *Brachypodium distachyon*; Bna, *Brassica napus*; Cas, *Camelina sativa*; Cme, *Cucumis melo*; Csa, *Cucumis sativus*; Csi, *Camellia sinensis*; Ghr, *Gossypium hirsutum*; Gma, *Glycine max*; Hvu, *Hordeum vulgare*; Lus, *Linum usitatissimum*; Mes, *Manihot esculenta*; Mtr, *Medicago truncatula*; Osa, *Oryza sativa*; Rco, *Ricinus communis*; Sbi, *Sorghum bicolor*; Stu, *Solanum tuberosum*; Zma, *Zea mays*; Pvi, *Panicum virgatum*.

**Figure 2:**
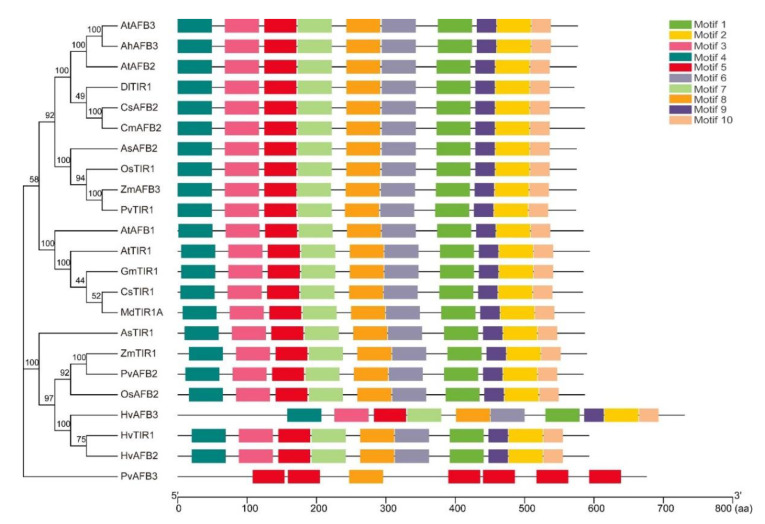
Phylogeny and structures of functionally characterized TIR1/AFBs in plants. Ah, *Arachis hypogaea*; At, *Arabidopsis thaliana*; As, *Agrostis stolonifera*; Cm, *Cucumis melo*; Cs: *Cucumis sativus*; Dl: *Dimocarpus longan*; Gm, *Glycine max*; Hv, *Hordeum* *vulgare*; Md, *Malus × domestica*; Os, *Oryza sativa*; Pv, *Panicum virgatum*; Zm, *Zea mays*.

Based on the submitted miRNA sequences in miRBase (https://mirbase.org/, accessed on 10 May 2022), we find that miR393 is a highly conserved miRNA family by sequence alignment, with 21–22 nt in eudicots and monocotyledonous plants, including model plants (e.g., *Arabidopsis thaliana*, *Medicago truncatula*), main crops such as rice (*Oryza sativa*), maize (*Zea mays*), soybean (*Glycine max*), cotton (*Gossypium hirsutum*), rapeseed (*Brassica napus*), camelina (*Camelina sativa*), sorghum (*Sorghum bicolor*) and their relatives (e.g., *Arabidopsis lyrata*, *Aegilops tauschii*, *Brachypodium distachyon*) (Figure 1). Only one or two nucleotide variations exist in the first or last two sites that are not the ‘seed’ region (critical miRNA-target pairing region) of mature miR393 [10,11,12]. There are also some miRNAs classified as miR393 which are uncertain, for example miR393b differs greatly from the other members in soybean [12]. The perfect, matched target genes of miR393 predicted by psRNATarget (https://www.zhaolab.org/psRNATarget/, accessed on 10 May 2022) are *TIR1* and *AFBs* (*AFB1/2/3*). Other genes, such as *bHLHs*, *GRR1-like protein 1* (*GRH1*), and *Arabidopsis Response Regulators* (*ARRs*), are also reported as miR393 targets with a single or more mismatches by bioinformatic or degradome analysis, but most of these have not been correlated with miR393 through biological function analysis [13,14]. Similar to the conservation of miR393, the TIR1 and AFBs are also highly conserved in the model plants and main crops (Figure 2).

miR393 members have distinctive expression patterns, and some of them are highly accumulated in the aerial organs of plants. For example, in *Arabidopsis*, miR393 was identified with greater abundance in leaves, stems, inflorescences and siliques, but was present at a very low level in roots [6]. Interestingly, miR393 was induced in *Arabidopsis* roots by nitrate, while the target genes *TIR1*, *AFB1*, *AFB2* and *bHLH77* were consistently repressed; however, *AFB3* was induced in response to nitrate [15]. Using promoter fusion lines, Parry et al. (2009) confirmed that miR393a and miR393b of *Arabidopsis* were expressed in primary root tips via *β*-glucuronidase (GUS) staining [7]. Similarly, the miR393b expression in rice was higher in aerial organs (e.g., leaves, shoots, booting panicles) but not expressed in roots, whereas miR393a was mainly expressed in the lateral root (LR) primordia of young seedlings and booting panicles [11]. The conserved targets of plant miR393s are the auxin receptors with F-box domains, TIR1 and AFBs, which function as components of the SKP/Cullin/F-box (SCF)-ubiquitin ligase complex and as Aux/IAA transcriptional repressors [16]. These auxin receptors associate with Cullin1 (CUL1) of SCF through the N-terminal H1 helix of the F-box domain [17]. Aux/IAAs recruit the TOPLESS (TPL) co-repressor to interact with both TPL and Auxin Response Factor (ARF) and, finally, repress the transcription of auxin-responsive genes [18]. Auxin is perceived by receptor complexes of TIR1/AFB and Aux/IAA, which could induce ubiquitination and 26S proteasome-mediated degradation of Aux/IAA, and release ARFs to activate auxin-responsive genes [19,20]. Thus, the miR393-TIR1/AFBs module is an important regulator that links auxin response with transcriptional regulation in plant development (Table 1).

## 3. Functional Diversity of miR393 and TIR1/AFBs in Plant Development

### 3.1. Leaf Development

In *Arabidopsis*, an *miR393b* mutant exhibited increased leaf number, elongated leaves, higher cotyledon epinasty and earlier senescence, indicating that miR393b was necessary for normal leaf development and auxin-regulated leaf morphology [6,9]. In addition, it was shown that miR393 was necessary for the biogenesis of siTAARs from *TAARs*. *miR393b* mutation increased the expression level of primary auxin-responsive genes *IAA1/12/19* and *DFL2*, which were important for leaf development via regulating *TAAR* expression, auxin perception and auxin signaling homeostasis [6,9]. This finding was in agreement with a previous report that mimicry of miR393 (MIM393) resulted in down-curled and narrow leaves in *Arabidopsis* [21]. The four F-box proteins TIR1, AFB1, AFB2 and AFB3 redundantly regulate diverse aspects of *Arabidopsis* growth and development, such as cotyledon number, leaf size and shape, hypocotyl and root length, and inflorescence height, by interacting with BDL (BODENLOS)/IAA12 and regulating auxin responses throughout plant development [22]. miR393a and miR393b were partially redundant for proper leaf morphogenesis in *Arabidopsis*; both single and double mutants exhibited complex changes in *AUX/IAA* and *GH3* gene expression [23]. In rice, miR393a/b overexpression increased the flag leaf inclination and affected root morphology and plant height. The overexpression plants were resistant to exogenous 2, 4-D, with insensitive root and shoot growth under high auxin conditions. Similar leaf inclination was observed in *OsTIR1*- and *OsAFB2*-suppressed lines [11]. This study also confirmed that OsTIR1 and OsAFB2 interacted with OsIAA1 to regulate ARF activity and auxin signal transduction [11]. In cucumber, miR393 repressed the post-transcriptional expression of *CsTIR1* and *CsAFB2*; furthermore, the overexpression of these target genes in tomato resulted in leaf curling and reduced stomata formation [48]. In barley, miR393 regulated seedling growth, stomatal density and guard cell length by affecting the auxin signaling pathway and stomata development-related gene expression [42].

### 3.2. Root Development

The miR393-TIR1/AFBs can control root development in plants. Using single, double, triple and quadruple mutants of *TIR1*, *AFB1*, *AFB2* and *AFB3*, Parry et al. (2009) found that *AFB3* was more important than *AFB1* in the auxin response of *Arabidopsis* roots. *TIR1*, *AFB2*, and *AFB3*, but not *AFB1*, exhibited post-transcriptional regulation by miR393. TIR1 and AFB2 are dominant auxin receptors in seedling roots, which have been shown to be negatively regulated by miR393 and could interact with Aux/IAA proteins to regulate lateral root formation [7]. In a further study, miR393 was induced with exogenous IAA treatment. Overexpressing the miR393-resistant form of TIR1 (mTIR1) enhanced auxin sensitivity, inhibited primary root growth but promoted lateral root growth in *Arabidopsis*. Interestingly, miR393 expression was promoted in *35S:mTIR1* plants via a feedback regulation [10]. miR393 has been shown to regulate the lateral root system by modulating TIR1, which could bind the IAA proteins [6]. Furthermore, the ectopic overexpression of *Arabidopsis* miR393a reduced plant size and root length in tobacco [54]. In rice, miR393a is expressed mainly in the roots, especially in crown root tips and lateral root primordia, whereas miR393b is expressed in aerial tissues. The overexpression of rice miR393a/b increased the primary root length and reduced the crown root number in rice, but the root morphologies of *OsTIR1*- and *OsAFB2*-RNAi lines were similar to that of the wild type [11]. Under the inhibition of NAA, Xia et al. (2012) observed more roots and longer main roots in Osa-miR393 overexpression plants than wild-type rice, but no difference was observed when grown in water [32]. miR393a promoted primary root elongation of rice seeds germinated in air but inhibited coleoptile elongation and stomatal development of submerged rice seeds via modulating the auxin signaling during seed germination and seedling establishment [33]. In a further study, Osa-miR393 overexpression was shown to reduce the lateral root number and plant sensitivity to IAA, via repressing Osa-miR390 and *OsTIR*. The regulation of Osa-miR393 on root development could be induced by ABA and various stresses (e.g., drought, salt, heavy metal) [34]. The mutation of OsTIR1 and OsAFB2-5 improved primary root length, decreased adventitious root (AR) number and lateral root density, through interacting with OsIAA1 and regulating auxin responsive genes *OsIAA9/20* [35]. Recently, Li et al. (2021) found that down-regulated miR393 and up-regulated AFB3 might contribute to the primary and lateral root growth of peanut under potassium (K) deficiency, while induced miR393 and repressed AFB3 expression were observed in roots under nitrogen (N) deficiency [47]. 

### 3.3. Root Nodulation

miR393 and target-module-regulated auxin signaling also play important roles in the root nodulation of legume plants. Mao et al. (2013) analyzed the roles of miR393 and miR160 in regulating nodule formation of soybean, and found that miR393 overexpression reduced plant sensitivity to auxin, and the level of auxin sensitivity required for indeterminate nodule formation was higher than that of determinate nodules [45]. In common bean, 28 up-regulated miRNAs have been identified as aluminum toxicity nodule-responsive miRNA, including miR393, miR164 and miR170 [55]. Cai et al. (2017) proved that *GmTIR1/AFB3* were post-transcriptionally cleaved by miR393, and the inhibition of miR393 and overexpression of mutated *GmTIR1C* at the cleavage site increased the nodule number of soybean [12]. Li et al. (2021) confirmed that miR393b of apple (*Malus* × *domestica* Borkh.) rootstock repressed the expression of *MdTIR1A* and negatively regulated the adventitious root formation and auxin sensitivity of tobacco [51]. 

### 3.4. Branching and Internode Growth

Branching and internode growth are important for plant height, yield and light perception [56]. Osa-miR393 overexpression was shown to repress the target genes (*OsTIR1* and *OsAFB2*), as well as an auxin transporter *OsAUX1* and a tillering inhibitor *OsTB1*, leading to increased tillers and early flowering in rice [32]. Osa-miR393 promoted rice tillering similar to the effect of nitrogen fertilizer, while Osa-miR393 mutation inhibited N-promoted tillering. The expression of OsIAA6 protein was positively regulated by Osa-miR393 and N nutrition, indicating that N-induced Osa-miR393 repressed *OsTIR1/AFB2*, alleviated axillary bud sensitivity to auxin and promoted tillering [36]. In rice, OsTIR1 and OsAFB2-5 could interact with OsIAA1 and redundantly regulate plant height and tillering number by affecting *OsIAA9/20* expression. The *Ostir1* and *Osafb2* mutants exhibited impaired plant height and increased tillering number compared to the wild type [35]. This finding was similar to a previous study that showed overexpression of miR393a/b reduced the plant height of rice [11]. However, the overexpression of miR393 target genes (*CsTIR1* and *CsAFB2*) from cucumber repressed the plant height of tomato [48]. Ectopic expression of Osa-miR393a in creeping bentgrass (*Agrostis stolonifera*) inhibited the expression of *AsAFB2* and *AsTIR1*, induced fewer but longer tillers, wider leaves and larger internodes [37]. Based on sRNA-seq and degradome analysis, Zhao et al. (2016) speculated that miR393 and the target genes might be involved in the internode elongation and development under maize ears [40]. The inhibition of miR393 in poplar was shown to promote phloem and xylem growth and increase lignin content through increasing the gene expression in the auxin signaling pathway, and the short tandem target mimic lines of miR393 (STTM393) showed more internodes and promoted growth compared with the control [57].

### 3.5. Flowering and Fruit Development

miR393 and its target genes can also control flowering, fruit and seed development. For example, in addition to the variations in root and leaf phenotype, the overexpression of mTIR1 also delayed the flowering of *Arabidopsis* [10]. miR393 was also shown to regulate silique length of *Arabidopsis* under different growth conditions, through affecting *ARF* and *IAA* expression and putative feedback regulation [6]. In rice, miR393 up-regulation led to early flowering, via repressing *OsTIR1* and *OsAFB2*, and auxin transporter *OsAUX1* [32]. OsTIR1 and OsAFB2 could interact with OsIAA1 to regulate auxin-responsive genes *OsIAA9/20*, and both *OsTIR1* and *OsAFB2* mutation impaired the grain number and germination of rice [35]. Bai et al. (2017) showed that miR393 up-regulated the length-width ratio of barley seeds, but both overexpression and mimicry of miR393 decreased the grain weight through targeting *TIR1/AFBs* in barley [43]. The overexpression of *CsTIR1* and *CsAFB2*, the target genes of miR393 in cucumber, impaired the seed size and seed germination in tomato [48]. The overexpression of Cme-miR393 repressed *CmAFB2* expression and delayed the fruit ripening of melon [52]. Shi et al. (2017) identified the miRNAs with reduced expression in peach fruit after NAA treatment, including miR393, miR156 and miR160. These miRNAs might control the fruit enlargement of peaches [58].

### 3.6. Secondary Metabolism and Other Roles

In additional to plant growth and development, the miR393/target gene module also plays a role in plant secondary metabolism, regenerability, and embryogenesis. Li et al. (2018) reported that blue light promoted the accumulation of flavonoids in embryogenic calli of longan, and miR393 expression was repressed, while *TIR1* was up-regulated under blue light [50]. Based on the profiling of the metabolome, transcriptome, degradome, and weighted correlation network analysis (WGCNA), Zhao et al. (2020) found that miR393-*bHLH* was negatively correlated with the biosynthesis of taste compounds (e.g., gallated catechin, caffeine, and theanine) in the tea plant (*Camellia sinensis*) [49]. miR393 inhibited the shoot regenerability and size of shoot apical meristem (SAM) in *Arabidopsis*, and similar regulation was shown in the *tir1-1* mutant [59]. Wojcik and Gaj (2016) proved that miR393 and TIR1/AFB controlled the somatic embryogenesis (SE) of *Arabidopsis* by affecting the explant sensitivity to auxin [24]. Omidvar et al. (2015) reported the miRNA expression pattern in a male-sterile tomato mutant and found that miR393 was down-regulated in the sterile line and might be involved in the anther development [25].

## 4. The Function of miR393 and TIR1/AFBs in Plant Abiotic Stress Response

### 4.1. Salt Stress

Salt stress induced miR393 expression in *Arabidopsis*, triggered stabilization of the Aux/IAA repressors and thus repressed the TIR1/AFB2-mediated auxin signaling. An *mir393ab* mutation enhanced lateral root number and root length during salinity, with an increased level of reactive oxygen species (ROS) and reduced ascorbate peroxidase (APX) activity compared to the wild genotype [26,27]. Chen et al. (2015) enhanced the salt tolerance of *A. thaliana* by overexpressing *mTIR1*, the transgenic lines displayed improved osmotic stress tolerance and accumulated more proline and anthocyanin. Compared with the wild type, the salt-stress-related genes were up-regulated, and the sodium content was reduced in mTIR1-overexpressing plants under salt stress [8]. Recently, Denver and Ullah (2019) found that the *mir393a*, *mir393b* and double mutant *mir393ab* of *Arabidopsis* were salt-sensitive, and miR393 regulated salt stress response through Receptor for Activated C Kinase 1A (RACK1A)-mediated ABA signaling [28]. Furthermore, the transgenic lines of tobacco overexpressing Ata-miR393a were less sensitive to IAA treatment and NaCl stress than the control [54]. Osa-miR393 expression changes under salt and alkali treatment, and the Osa-miR393 overexpression plants of rice and *Arabidopsis* were more sensitive to salinity and alkaline stresses [38]. In *Triticum aestivum*, miR393 was dramatically down-regulated under wounding treatment but was induced by salt stress [60].

### 4.2. Drought and Waterlogging

In plants, miR393 expression can be regulated by abiotic stresses, such as drought and waterlogging [61]. The lateral root growth of *Arabidopsis* overexpressing miR393-resistant AFB2 and TIR1 were found to be insensitive to ABA and osmotic stress, but the LRs in wild-type were significantly inhibited under ABA and PEG treatment. This indicated that miR393 inhibited LR development and regulated plant drought resistance by targeting *TIR1/AFB2* [29]. In addition to the roles in controlling tillering and flowering, Osa-miR393 could negatively regulate plant tolerance to salt and drought stresses, as well as plant sensitivity to auxin [32]. Lu et al. (2018) also reported that Osa-miR393 overexpression reduced the LR number and sensitivity to IAA by repressing Osa-miR390 and *OsTIR*. This regulation of Osa-miR393 on root development could be induced by ABA and various stresses (e.g., drought, salt, heavy metal) [34]. In maize, miR393 is a regulator in root development under short-term waterlogging, which could be induced under stress conditions in a waterlogging tolerant line but inhibited in a sensitive line [41]. In *Cynara cardunculus*, De Paola et al. (2012) found that Cca-miR393a was slightly up-regulated in salt-stressed leaves of globe artichoke, but not in the roots [62]. miR393 overexpression in barley enhanced drought sensitivity, while the knockdown of miR393 improved drought tolerance; this might be regulated through the ABA pathway [42]. miR393 was also down-regulated in different genotypes of *Sorghum bilolor* under drought stress [63]. The miR393 expression in wild *Ipomoea campanulata* and cultivated *Jacquemontia pentantha* was repressed under water deficit, but it was up-regulated in drought-stressed *Arabidopsis* [64]. In tomato, Sly-miR393 was up-regulated in above ground tissues of a drought-tolerant genotype and down-regulated in roots of a drought-sensitive genotype [14].

### 4.3. Temperature Stress

miR393 expression in *Arabidopsis* has also been shown to be induced by cold stress; *TIR1* was inhibited in response to low temperature [61]. Osa-miR393 could also regulate abiotic stress response of other monocotyledonous plants. For instance, Osa-miR393 ectopic expression improved the cold tolerance and tillering of switchgrass (*Panicum virgatum*). The cold responsive genes (*PvCOR47*, *PvICE1* and *PvRAV1*) were up-regulated, and the biomass and soluble sugar content were also increased in transgenic plants [53]. This study also showed that Pvi-miR393 of switchgrass was up-regulated by cold stress and down-regulated by auxin, while the predicted target genes (*PvAFB1*, *PvAFB2*, *PvAFB3* and *PvTIR1*) were induced by cold stress but in different patterns [53]. The overexpression of Osa-miR393a in creeping bentgrass improved plant tolerance to salt, drought and heat stresses [37]. In wheat, miR393 could be up-regulated by salt and osmotic stresses but down-regulated under cold stress [65]. In banana, miR393-TIR1/AFB-triggered phasiRNAs were specifically enriched under cold stress [66]. After high-temperature (HT) treatment, Ghr-miR393 was up-regulated, but Ghr-novel-miR393b-3p and Ghr-novel-miR393c-3p were down-regulated in a HT-tolerant line of cotton compared with a HT-sensitive line [67].

### 4.4. Nutritional Stresses

In *Arabidopsis*, the miR393/AFB3 module controlled the root system architecture (RSA) in response to nitrate supply. miR393 and *AFB3* expression in roots were consistently regulated by nitrate, and *AFB3* could be induced by nitrate but then repressed by N metabolites from nitrate reduction and assimilation. Unlike the inhibited root development in wild type, both miR393-overexpression and *AFB3* mutation increased the primary root length in seedlings under three days of KNO_3_ (5 mM) treatment [15]. Li et al. (2016) reported that nitrogen induced up-regulation of Osa-miR393 and down-regulation of target genes (*OsAFB2* and *OsTB1*) [36]. In peanut, miR393 and AFB3 expression were regulated by potassium and nitrogen deficiency, thus affecting the primary and lateral root growth [47]. Song et al. (2015) found that miR393 was up-regulated in leaves of *Chrysanthemum nankingense* under low-nitrogen conditions [68]. Lu et al. (2015) reported that miR393 was up-regulated in boron-deficient leaves of *Citrus sinensis* compared to the control, which might affect plant growth and development by repressing auxin signaling due to repressed TIR1 and AFB1/2 [69]. Under nitrogen stress, miR393 and the target genes were regulated coordinately in an N-stressed sensitive *Populus* clone (*Nanlin 895*) [70].

### 4.5. Metal Stresses

It has been speculated that miR393 has functions in plant response to metal stresses. He et al. (2014) reviewed the aluminum-responsive miRNAs in plants, including miR393 that was up-regulated in *M. truncatula* exposed to Al [71,72]. Ding and Zhu (2009) reviewed the miRNAs involved in plant adaptive response to copper and cadmium (Cd) stresses, of which, miR393 functioned in relieving Cd stress by repressing the target genes, *TIR1* and *bHLHs*, in *M. truncatula*, *Brassica napus* and rice [71,73,74,75]. In leaves, miR393 could be induced by metals such as Cd, hydrargyrum (Hg) and Al, and might play a role as a regulator in plant response to metal toxicity [71,76]. miR393 was up-regulated in 24 h aluminum toxicity roots from nitrate-fertilized common beans (*Phaseolus vulgaris*) [55]. Dmitriev et al. (2017) first reported that miR393, miR390 and miR319 were response to aluminum stress in flax, among which miR393 was up-regulated in resistant cultivars under Al stress [77]. The miR393 expression in the root apex of barley was inhibited by aluminum stress; the overexpression of miR393 alleviated the Al-induced root inhibition and reactive oxygen species (ROS)-induced cell death, while the inhibited miR393 expression enhanced the root sensitivity to Al stress [44].

## 5. The Role of miR393 and TIR1/AFBs in Biotic Stress Response

In *Arabidopsis*, the complementary strand of miR393 (miR393b*) has been identified as an Argonaute 2 (AGO2)-bound sRNA, which could target *MEMB12* encoding a SNARE protein localized in Golgi apparatus. *Pseudomonas syringae* pv. *tomato* (*Pst*) infection induced AGO2 and inhibited MEMB12 in a miR393b*-dependent manner. Both miR393b* overexpression and *memb12* mutation promoted the secretion of PR1, an antimicrobial pathogenesis-related protein in *Arabidopsis*, indicating that AGO2 and miR393/MEMB12 are important effectors or regulators in plant antibacterial immunity [30]. Ath-miR393 was induced during pattern-triggered immunity, and miR393 overexpression suppressed auxin signaling and inactivated ARF1/9 expression, increasing glucosinolate and decreasing camalexin levels, which were related to the plant resistance to biotrophic pathogens and susceptibility to necrotrophic pathogens, respectively. AtAFB1 overexpression could prevent inhibited auxin signaling by flg22, then reduce salicylic acid accumulation and cause plant susceptibility to biotrophs [78]. Zhao et al. (2012) isolated AGO-associated sRNA in *Arabidopsis* using immune precipitation and deep-sequencing, showing that miR393 was specifically enriched in bacterial-challenged plants [79]. The inoculation of *Burkholderia phytofirmans* PsJN on *A. thaliana* overexpressing miR393 did not increase the primary root length, fresh weight, or total chlorophyll content compared to the wild type. PsJN inoculation could not affect the target gene expression of miR393 in the wild *A. thaliana* at the four-leaf stage, but *AFB1/3* were up-regulated in the inoculated plants at the six-leaf stage [80]. Djami-Tchatchou and Dubery (2019) screened the miRNA expression pattern of bacterial lipopolysaccharide (LPS)-treated *Arabidopsis* leaf and callus, finding miR393 was repressed, but the target gene *LecRLK* was up-regulated, which might be related to the enhanced perception ability of LPS in *Arabidopsis* [81]. The overexpression and repression of miR393, respectively, suppressed and induced the expression of *Lectin Receptor-Like Kinases* (*LecRLK*) in *Arabidopsis* treated with LPS [82]. Shi et al. (2015) proved that miR393-mediated auxin signaling was involved in the hydrogen sulfide (H_2_S)-mediated antibacterial resistance of *Arabidopsis* [31]. Sulfur dioxide (SO_2_) pre-exposure of *Arabidopsis* resulted in the up-regulation of miR160, miR167 and miR393 and enhanced disease resistance against *Botrytis cinerea* [83]. 

miR393 can also control the biotic stress response in crops. In tobacco, miR393 was induced in leaves infiltrated with an oncogenic strain of *Agrobacterium tumefaciens* (C58), which may contribute to plant antibacterial resistance via repressing auxin signaling [84]. Recently, Nazari et al. (2017) found that miRNAs (miR393 and miR167) and flavonoids could be taken as biomarkers in the tobacco–*Agrobacterium* interaction, which were up-regulated or accumulated when treated with *Bacillus subtilis* [85]. In soybean, miR393 was up-regulated by soybean mosaic virus (SMV) infection, and the suppression of genes in auxin signaling pathways might be related to plant defense responses [86]. Supported with sRNA-seq data, Xu et al. (2015) found that miR393 was up-regulated in a soybean cyst nematode (SCN)-resistant line compared to a susceptible line of soybean, which exclusively target genes encoding TIR1, AFBs and ribosomal protein L20 [87]. Additionally, miR393 was also induced by *Phytophthora sojae* infection, knockdown of miR393 enhanced plant susceptibility to *P. sojae*, accompanied with repressed isoflavonoid biosynthetic gene expression in roots [46]. miR393 was a negative regulator of arbuscule formation in *O. sativa*, *S. lycopersicum*, and *M. truncatula*, by inhibiting the auxin receptor genes (*TIR1* and *AFBs*) and auxin perception in arbuscule-containing cells [88]. In rice, the overexpression of miR393 decreased the *TIR1* expression and increased plant susceptibility to Rice Black Streaked Dwarf Virus (RBSDV) [39]. sRNA-seq analysis revealed that the down-regulation of miR393 in maize might be related to the plant antiviral defense to synergistic infection [89]. The miR393 expression was significantly decreased in a sensitive cultivar (Hanatee) of cassava infected by *Colletotrichum gloeosporioides* but increased in resistant cultivar Huay Bong 60 [90]. 

Recently, miR393 was reported with expressional changes in fruits and vegetables under biotic stresses. In mulberry, miR393A was induced by phytoplasma infection, but miR393B was unaltered in infected leaves, and the two miR393 were regulated by different *cis*-acting elements [91]. Chand et al. (2017) identified 45 miRNAs responsive to immunity in garlic (*Allium sativum*), and transgenic plants overexpressing miR393, miR164a and miR168a showed enhanced resistance to *Fusarium oxysporum* f. sp. *cepae* [92]. Vinutha et al. (2020) found miR393 was up-regulated in tomato infected with Leaf Curl New Delhi Virus (ToLCNDV) [93].

## 6. Conclusions

As a miRNA family with conserved biological functions in plants, miR393 and its target genes TIR1/AFBs are broadly involved in the growth and development of leaf, root, branching, seed, secondary metabolism, as well as abiotic (e.g., salt, drought, cold, heavy metal) and biotic stress responses. So far, the regulatory network and putative applications of miR393/target module has been elaborated in *Arabidopsis*, rice, soybean, barley and cucumber. miR393s in these plants regulate auxin perception and signaling, Aux/IAA degradation and auxin-responsive gene expression, mainly by repressing auxin receptors TIR1 and AFBs. The miR393-TIR1/AFBs module is an important regulator links auxin response with transcriptional regulation in plant development; thus, we can harness this module as a valuable tool to manipulate crop traits for optimized yield and adaptability. This module may also be important in controlling development, agronomic trait, environmental adaptation of other plants such as tomato, tobacco, wheat and rapeseed, but the molecular mechanism of miR393/targets in these plants were barely reported except for the expressional variations under different growth conditions. On the other hand, it is still unclear whether there are other co-effectors or regulators of TIR1/AFBs, in addition to Aux/IAAs and CUL1. It is also intriguing that TIR1 and AFBs have different abilities to interact with IAAs and influence SCF assembly. For example, AFB1 in *Arabidopsis*, specialized later than other receptors, may play a unique role in Brassicaceae [94]. Additionally, the evolution and function of different miR393 members in crops remain unclear; many speculated members reported from degradome sequencing were not validated. How these miR393s and target genes regulate plant development and adaptation are unclear. A recent study showed that TIR1 and AFB2 were positive modulators of jasmonic acid (JA) homeostasis and AR formation in *Arabidopsis*, through controlling JA biosynthesis and conjunction [19]. This suggested that miR393-TIR1/AFBs might be involved in other hormone signaling pathways, and it is innovative to establish a connection between JA and miR393-auxin pathways. A detailed understanding of miR393 and different target genes will facilitate the design and utilization of this module in precise modification of agronomic traits and stress resistance in crops.

## Figures and Tables

**Table 1:** The functionally characterized miR393 and TIR1/AFBs in plants.

Plant Species	miRNA	Target Genes	Interactive Proteins	Regulated Genes	Functions	References
*Arabidopsis thaliana*	miR393a miR393b	*AtTIR1* *AtAFB1* *AtAFB2* *AtAFB3* *AtGRH1*	BDL/IAA12	*IAA1/3/7/12/19*, *GH3*, *DFL2*, *ARF1/9*	Regulates somatic embryogenesis, leaf shape and number, cotyledon epinasty, senescence, hypocotyl and root length, inflorescence height, and primary root length in response to nitrate. This module involves in hydrogen sulfide-mediated antibacterial resistance and immunity to *Pseudomonas syringae* pv. *tomato*. It also regulates glucosinolate and camalexin level to involve in plant resistance to biotroph and necrotroph pathogens. It regulates salt and osmotic stress responses through ABA signaling.	[6,7,8,9,10,15,21,22,23,24,25,26,27,28,29,30,31]
*Oryza sativa*	miR393a miR393b	*OsTIR1 OsAFB2*	IAA1	*ARF*, *IAA6/9/20*, *miR390*, *AUX1*, *TB1*	Regulates tillering, flag leaf inclination, flowering and sensitivity to 2,4-D, primary and crown root growth, plant height, coleoptile elongation and stomatal development of submerged seeds, grain number and seed germination. The module is regulated by nitrogen and affects nitrogen-promoted tillering. It also plays roles in plant tolerance to salt, alkaline and drought stresses, as well as plant immunity to rice black streaked dwarf virus.	[11,32,33,34,35,36,37,38,39]
*Zea mays*	miR393	*ZmTIR1* *ZmAFB*			Might be involved in the internode elongation and development under maize ears; acts as a regulator in root development under short-term waterlogging.	[40,41]
*Hordeum vulgare*	miR393	*HvTIR1 HvAFB2 HvAFB3*		*ARF5*, *EPF1*, *SPCH*, *MUTE*	Regulates seedling growth, stomatal density and guard cell length. miR393 positively regulates length–width ratio of seeds and grain weight, and negatively regulates drought tolerance of barley. miR393 up-regulation alleviated aluminum-induced root inhibition and ROS-induced cell death, its down-regulation enhanced root sensitivity to aluminum stress.	[42,43,44]
*Glycine max*	miR393				Regulates root development and nodule formation of soybean and alfalfa. Inhibition of Gma-miR393 and overexpression of mutated GmTIR1C at the cleavage site increased the nodule number of soybean. Knockdown of Gma-miR393 enhanced plant susceptibility to *Phytophthora sojae*, repressed isoflavonoid biosynthetic gene expression in roots.	[12,45,46]
*Arachis hypogaea*	miR393	*AhAFB3*			Regulated by potassium and nitrogen deficiency, affects the primary and lateral root growth under nutrient deficiency.	[47]
*Cucumis sativus*	miR393	*CsTIR1* *CsAFB2*			CsTIR1 and CsAFB2 overexpression caused curling leaf and reduced stomata, poor seed germination, reduced plant height and seed size in tomato.	[48]
*Camellia sinensis*	miR393	*CsbHLH*			Negatively correlated with the biosynthesis of taste compounds, gallated catechin, caffeine, and theanine.	[49]
*Dimocarpus longan*	miR393	*DlTIR1*			miR393 was repressed and TIR1 was up-regulated under blue light.	[50]
*Malus × domestica*	miR393b	*MdTIR1A*			Negatively regulates adventitious root formation and auxin sensitivity of tobacco.	[51]
*Cucumis melo*	miR393	*CmAFB2*			miR393 overexpression delayed fruit ripening.	[52]
*Agrostis stolonifera*	miR393a	*AsAFB2* *AsTIR1*			Osa-miR393a inhibited AsAFB2/AsTIR1 expression; induced fewer and longer tillers, wider leaves and larger internodes; and improved plant tolerance to salt, drought and heat stresses.	[37]
*Panicum virgatum*	miR393	*PvAFB2* *PvAFB3 PvTIR1*		*PvCOR47*, *PvICE1*, *PvRAV1*	Osa-miR393 improved cold tolerance and tillering of switchgrass. Pvi-miR393 was up-regulated by cold stress and down-regulated by auxin, the target genes were induced by cold stress with different patterns.	[53]

## Data Availability

Not applicable.
